# DPP-4 inhibitor and alpha-glucosidase inhibitor equally improve endothelial function in patients with type 2 diabetes: EDGE study

**DOI:** 10.1186/s12933-014-0110-2

**Published:** 2014-07-30

**Authors:** Kazufumi Nakamura, Hiroki Oe, Hajime Kihara, Kenei Shimada, Shota Fukuda, Kyoko Watanabe, Tsutomu Takagi, Kei Yunoki, Toru Miyoshi, Kumiko Hirata, Junichi Yoshikawa, Hiroshi Ito

**Affiliations:** Department of Cardiovascular Medicine, Okayama University Graduate School of Medicine, Dentistry and Pharmaceutical Sciences, 2-5-1 Shikata-cho, Okayama, 700-8558 Japan; Department of Internal Medicine, Kihara Cardiovascular Clinic, Asahikawa, Japan; Department of Internal Medicine and Cardiology, Osaka City University of Medicine, Osaka, Japan; Department of Medicine, Osaka Ekisaikai Hospital, Osaka, Japan; Department of Internal Medicine, Okayama Saiseikai General Hospital, Okayama, Japan; Takagi Cardiology Clinic, Kyoto, Japan; Department of Cardiovascular Medicine, Wakayama Medical University, Wakayama, Japan; Nishinomiya Watanabe Cardiovascular Center, Nishinomiya, Japan

**Keywords:** Dipeptidyl peptidase 4 (DPP-4) inhibitors, Alpha glucosidase inhibitor, Endothelial function, Flow-mediated dilatation, CD34

## Abstract

**Background:**

Alpha glucosidase inhibitor (GI) attenuates postprandial hyperglycemia (PPH) and reduces the risk of cardiovascular events in patients with impaired glucose tolerance or type 2 diabetes. Dipeptidyl peptidase 4 (DPP-4) inhibitors also attenuate PPH. PPH is one of the factors leading to endothelial dysfunction which is an early event in the pathogenesis of atherosclerosis. Furthermore, DPP-4 inhibitors protect endothelial function through a GLP-1-dependent mechanism. However, the impact of these two types of drugs on endothelial dysfunction in patients with type 2 diabetes has not been fully elucidated. We compared the effects of sitagliptin, a DPP-4 inhibitor, and voglibose, an alpha GI, on endothelial function in patients with diabetes.

**Methods:**

We conducted a randomized prospective multicenter study in 66 patients with type 2 diabetes who did not achieve the treatment goal with sulfonylurea, metformin or pioglitazone treatment; 31 patients received sitagliptin treatment and 35 patients, voglibose treatment. The flow-mediated dilatation (FMD) of the brachial artery was measured in the fasting state at baseline and after 12 weeks of treatment. The primary endpoint was a change in FMD (ΔFMD) from the baseline to the end of follow-up. The effects of sitagliptin and voglibose on FMD were assessed by ANCOVA after adjustment for the baseline FMD, age, sex, current smoking, diabetes duration and body mass index. Secondary efficacy measures included changes in HbA1c, GIP, GLP-1, C-peptide, CD34, lipid profile, oxidative stress markers, inflammatory markers and eGFR and any adverse events.

**Results:**

ΔFMD was significantly improved after 12 weeks of treatment in both groups, and there was no significant difference in ΔFMD between the two groups. There were no significant differences in changes in HbA1c, GIP, GLP-1, C-peptide, lipid profile, oxidative stress marker, inflammatory marker and eGFR between the two groups. Compared with voglibose, sitagliptin significantly increased the circulating CD34, a marker of endothelial progenitor cells. Adverse events were observed in 5 patients in only the voglibose group (diarrhea 1, nausea 1, edema 2 and abdominal fullness 1).

**Conclusions:**

Sitagliptin improved endothelial dysfunction just as well as voglibose in patients with type 2 diabetes. Sitagliptin had protective effects on endothelial function without adverse events.

**Trial registration:**

registered at http://www.umin.ac.jp/ctrj/ under UMIN000003951

## Introduction

Postprandial hyperglycemia (PPH) plays a major role in cardiovascular complications in patients with type 2 diabetes [1] and impaired glucose tolerance (IGT) [2]. PPH is one of the main factors leading to endothelial dysfunction, which is an early event in the pathogenesis of atherosclerosis [3,4].

Alpha glucosidase inhibitor (GI) prevents the digestion of carbohydrates including starch and table sugar, attenuates postprandial hyperglycemia [5] and delays the development of type 2 diabetes in patients with IGT [6]. Miglitol, an alpha GI, improves endothelial dysfunction assessed by the response of forearm blood flow to reactive hyperemia and flow-mediated dilatation (FMD) in patients with type 2 diabetes and coronary artery disease [7,8]. Acarbose, an alpha GI, improves postprandial endothelial dysfunction in patients with type 2 diabetes [9,10] and reduces the risk of cardiovascular events in patients with type 2 diabetes [11] and IGT [12,13].

Dipeptidyl peptidase 4 (DPP-4) inhibitor enhances endogenous incretin action and promotes glucose-dependent insulin secretion. Thus, DPP-4 inhibitor attenuates postprandial hyperglycemia [14]. Furthermore, glucagon-like peptide-1 (GLP-1), an incretin, induces an endothelial-dependent relaxation via NO-dependent action [15] and improves endothelial dysfunction in patients with type 2 diabetes [16], and sitagliptin, a DPP-4 inhibitor, protects endothelial function in spontaneously hypertensive rats through a GLP-1-dependent mechanism [17]. However, the impact of these two types of drugs on endothelial dysfunction in patients with type 2 diabetes has not been fully elucidated and has been controversial [18,19].

We conducted a randomized prospective multicenter study to compare the effects of sitagliptin, a DPP-4 inhibitor, and voglibose, an alpha GI, on endothelial function assessed by FMD in patients with type 2 diabetes.

Liao et al. reported that number of circulating endothelial progenitor cells (EPCs) in patients with type 2 diabetes was significantly lower than that in the healthy subjects, treatment with metformin significantly increased EPCs and the EPCs number was related to endothelial function assessed by FMD [20]. Fadini et al. reported that sitagliptin increased circulating EPCs in type 2 diabetic patients [21]. We also compared the effects of sitagliptin and voglibose on number of circulating EPCs assessed by measurement of CD34, a maker of EPCs [22], postive cells in patients with type 2 diabetes in this study.

DPP-4 inhibitors have anti-inflammatory and anti-oxidative effects [23-25]. Ishibashi et al. reported that linagliptin inhibited the generation of reactive oxygen species induced by advanced glycation end products (AGEs) in endothelial cells [23]. Matsubara et al. reported that sitagliptin improves endothelial dysfunction in association with ant-inflammatory effects in patients with coronary artery disease and uncontrolled diabetes [24]. Shiraki et al. reported that GLP-1 reduced TNF-α-induced oxidative stress in endothelial cells [25]. We also measured levels of inflammatory markers, including high-sensitivity C-reactive protein (hs-CRP) and pentraxin-3 (PTX-3), and oxidative stress markers, including malondialdehyde-modified low-density lipoprotein (MDA-LDL) and urine 8-hydroxy-2’-deoxyguanosine (8-OHdG).

The primary endpoint in this study was a sitagliptin- or voglibose-induced change in FMD (ΔFMD) from baseline to the end of follow-up. Secondary efficacy measures included changes in HbA1c, gastric inhibitory peptide (GIP), GLP-1, C-peptide, CD34, lipid profile, adiponectin, oxidative stress markers including MDA-LDL and urine 8-OHdG, inflammatory markers including hs-CRP and PTX-3, and estimated glomerular filtration rate (eGFR) and any adverse events.

## Methods

### Study populations

We conducted a randomized prospective multicenter study in patients with type 2 diabetes who did not achieve the treatment goal with diet, exercise, sulfonylurea, metformin or pioglitazone treatment. We recruited 66 patients (men and women) who were from 20 to 85 years of age. Thirty-one patients received the sitagliptin (50 mg/day) treatment and 35 patients, the voglibose (0.6 mg/day) treatment. The doses of the two drugs used in this study are recommended therapeutic doses for Japanese [26,27] and the doses are covered by the Japanese National Health Insurance. The exclusion criteria were as follows: treatment with insulin, alpha GI or glinide, type 1 diabetes, HbA1c ≥ 9.0%, systolic blood pressure ≥ 160 mmHg and serum creatinine ≥ 1.5 mg/dL at baseline. The study protocol was approved by the Ethics Committee of Okayama University Graduate School of Medicine, Dentistry, and Pharmaceutical Sciences, and written informed consent was obtained from all patients before any study procedure was undertaken.

### Study protocol

**Figure 1:**
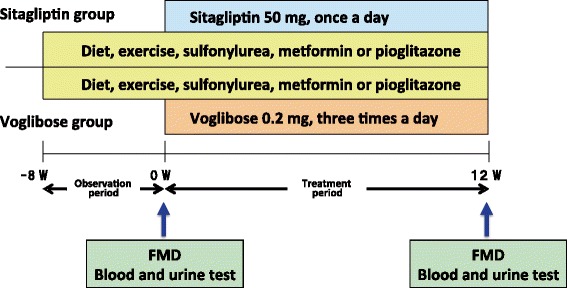
**Study protocol.** The patients were followed for at least 8 weeks to confirm that they did not achieve the treatment goal. The patients were prospectively, randomly assigned to additional treatment with either sitagliptin (50 mg/day) or voglibose (0.6 mg/day) for 12 weeks. Measurements of flow-mediated dilatation (FMD) of the brachial artery and blood and urine tests were performed in the fasting state at baseline and after 12 weeks of treatment.

The patients were followed for at least 8 weeks to confirm that they did not achieve the treatment goal with diet, exercise, sulfonylurea, metformin or pioglitazone treatment. The patients were prospectively randomly assigned to additional treatment with either sitagliptin (50 mg/day) or voglibose (0.6 mg/day) for 12 weeks (Figure 1). The flow-mediated dilatation (FMD) of the brachial artery was measured in the fasting state at baseline and after 12 weeks of the treatment. Blood and urine tests were also performed at baseline and at the end of the study. The patients’ antihypertensive, antihyperlipidemic and antidiabetic drugs were not changed and anti-oxidant drugs, including vitamin C and E, were not added throughout the study period.

### Measurements of biochemical parameters

The following parameters were measured at baseline and after 12 weeks of treatment: complete blood count, liver function test including measurement of AST, ALT and LDH, renal function test including measurement of BUN, creatinine, Na, K and Cl, HbA1c, gastric inhibitory peptide (GIP), GLP-1, C-peptide, CD34, lipid profile including total cholesterol, triglyceride, and high-density lipoprotein (HDL-C), adiponectin, oxidative stress markers including MDA-LDL and urine 8-OHdG, inflammatory markers including hs-CRP and PTX-3, and estimated glomerular filtration rate (eGFR). HbA1c levels were measured using high-performance liquid chromatography. The number of CD34+ cells was determined by flow cytometry using FITC-labeled CD45 and phycoerythrin (PE)-labeled CD34 antibodies (BD Biosciences). eGFR (mL/min/1.73 m^2^) was determined by the modified Modification of Diet and Renal Disease study formula (MDRD) for Japanese: eGFR = 194 × (age^-0.287^) × (serum creatinine^-1.094^) × (0.739 if female) [28]. These measurements were performed by SRL Company, Ltd. (Tokyo, Japan).

### Flow-mediated dilation (FMD)

Endothelium-dependent dilation was assessed as a parameter of vasodilation according to the guidelines for ultrasound assessment of FMD of the brachial artery in the fasting state [29]. Using a 10-MHz linear-array transducer probe (Unex Company Ltd., Nagoya, Japan), longitudinal images of the brachial artery at baseline were recorded with a stereotactic arm, and measurements of the artery diameter were made after supine rest for ≥5 min as previously described [30-32]. The diameter of the artery was measured from clear anterior (media-adventitia) and posterior (intima-media) interfaces, which were manually determined. Then, suprasystolic compression (50 mmHg higher than systolic blood pressure) was performed at the right forearm for 5 min, and measurements of the artery diameter were made continuously from 30 sec before to ≥2 min after cuff release. Maximum vasodilation was then evaluated from the change in artery diameter after the release of occlusion (%FMD). FMD is known to be affected by a wide range of biological, environmental, and methodological factors [33]. To quantify inter- and intra-observer reproducibility, baseline brachial diameter and FMD were measured by three individuals in Okayama University Hospital. Inter- and intra-observer coefficients were high (r > 0.90).

### Statistical analysis

The results are expressed as mean ± SD. We assumed that FMD increased by 2% in sitagliptin group and decreased by 0.5% in voglibose group with a standard deviation of 3%. A minimum sample size of 24 participants in each group was required to detect statistical differences in FMD with a power of 80% and α error of 5%. The effects of sitagliptin and voglibose on FMD were assessed by ANCOVA after adjustment for the baseline FMD, age, sex, current smoking, diabetes duration and body mass index (BMI). Differences in age, sex, BMI and abdominal girth were compared using Student’s t-test. The duration of diabetes was compared using the Wilcoxon rank sum test and categorical variables, using Fisher’s exact test. Differences in secondary efficacy measures between baseline and 12 weeks were compared using the paired Student’s t-test. Values of P < 0.05 were considered to be statistically significant.

## Results

### Baseline characteristics

Although 66 patients with type 2 diabetes were enrolled in this study between September 2010 and December 2011, 6 patients were excluded from this study (4 protocol violation at baseline and 2 withdrawal of consent). During the follow-up period, 5 patients with hypertension dropped out of the study due to change of concomitant antihypertensive drugs. The follow-up study was completed in 55 (83%) of the patients; 24 patients received the sitagliptin treatment and 31 patients, the voglibose treatment. The data collection was completed in July 2012. Baseline characteristics, including age, sex, BMI and the type of anti-diabetic medication are shown in Table 1. It has been reported that endothelium-dependent vasodilation assessed by FMD declines with advance of age [34,35]. We recruited men and women who were from 20 to 85 years of age. The age range was quite wide in our study, but there was no significant difference in age or baseline FMD between the two groups (age, sitagliptin: 66.6 ± 11.9, >70 years: 12, median 69.5, range: 37 – 84 versus voglibose: 68.4 ± 9.2, >70 years: 14, median 70.0, range: 48 – 80 years old, P = 0.529, Table 1) (baseline FMD, sitagliptin: 5.41 ± 2.25 versus voglibose: 4.96 ± 2.16%, P = 0.450, Table 2). We did not exclude patients with severe dyslipidemia, but there were no significant differences in use of antihyperlipidemic drugs (Table 1) and baseline total cholesterol, triglyceride and HDL-C (Table 3) between the two groups. No patients died, developed cardiovascular events or were admitted to the hospital during the course of the study.

**Table 1:** **Baseline clinical characteristics**

**Variables**	**Sitagliptin, n = 24**	**Voglibose, n = 31**	**P value**
Age, y	66.6 ± 11.9	68.4 ± 9.2	0.529
Male	10 (41.7%)	18 (58.1%)	1.000
Diabetes duration, m	57.6 ± 41.2	41.9 ± 44.1	0.075
BMI, kg/m^2^	27.8 ± 3.5	25.7 ± 4.3	0.061
Abdominal girth, cm	91.4 ± 11.1	85.6 ± 17.4	0.303
Current smoking	5 (20.8%)	5 (16.1%)	0.475
Regular alcohol drinkers	4 (16.7%)	9 (29.0%)	0.349
Diabetic complication	1 (4.2%)	2 (6.5%)	1.000
Diabetic retinopathy	1 (4.2%)	0 (0%)	0.436
Diabetic nephropathy	0 (0%)	2 (6.5%)	0.499
Diabetic neuropathy	0 (0%)	0 (0%)	-
Hypertension	20 (83.3%)	25 (80.6%)	1.000
Hyperlipidemia	15 (62.5%)	18 (58.1%)	0.787
Hyperuricemia	4 (16.7%)	2 (6.5%)	0.387
Renal disturbance	3 (12.5%)	2 (3.2%)	0.307
Established Cardiovascular diseases	7 (29.2)	7 (22.6)	0.756
Cerebrovascular disease	4 (16.7%)	1 (3.2%)	0.156
Myocardial infarction	3 (12.5%)	5 (16.1%)	1.000
Peripheral artery disease	2 (8.3%)	1 (3.2%)	0.575
Antidiabetic drugs			
Pioglitazone	11 (45.8)	16 (51.6%)	0.787
Sulfonylurea	5 (20.8%)	3(9.7%)	0.276
Metformin	3 (12.5%)	0 (0%)	0.077
Antihypertensive drugs			
ARB	15 (62.5%)	19 (61.3%)	1.000
CCB	13 (54.2%)	18 (58.1%)	0.791
Diuretics	9 (37.5%)	10 (32.3%)	0.778
Others	11 (45.8%)	12 (38.7%)	0.783
ACE-I	1 (4.2%)	3 (9.7%)	
α-Blocker	4 (16.7%)	3 (9.7%)	
β-Blocker	3 (12.5%)	5 (16.1%)	
αβ-Blocker	1 (4.2%)	1 (3.2%)	
Aldosterone antagonist	2 (8.3%)	0 (0%)	
Antihyperlipidemic drugs	2 (8.3%)	0 (0%)	
Statins	12 (50.0%)	14 (45.2%)	0.789
Fibrate	2 (8.3%)	2 (6.5%)	1.000
Ezetimibe	3 (12.5%)	6 (19.4%)	0.716
Eicosapentaenoic acid	2 (8.3%)	3 (9.7%)	1.000
Antithrombogenic agents			
Antiplatelet agent	16 (66.7%)	17 (54.8%)	0.417
Anticoagulant agent	2 (8.3%)	0 (0.0%)	0.186
Others			
Nitrates	3 (12.5%)	0 (0%)	0.077
Allopurinol	1 (4.2%)	1 (3.2%)	1.000
Uricosuric agents	0 (0%)	1 (3.2%)	1.000

ARB: angiotensin receptor blocker, CCB: calcium channel blocker, ACE-I: Angiotensin-converting enzyme inhibitor. Data are expressed as mean ± SD or as a number (percentage).

**Table 2:** **Changes in FMD (ΔFMD) in the sitagliptin and voglibose group**

**Variable**	**Sitagliptin**	**Voglibose**	**Between-group difference**
	**n = 24**	**n = 31**	
FMD (%)			
Baseline at 0 W	5.41 ± 2.25	4.96 ± 2.16	P = 0.450
at 12 W	6.17 ± 2.00	5.94 ± 2.15	P = 0.692
ΔFMD:12 W-0 W	0.76 ± 2.42	0.98 ± 2.41	P = 0.729
Adjusted ΔFMD:12 W-0 W*	1.11	0.98	P = 0.8316
(95% CIs)	(0.07-2.16)	(0.04-1.91)	

Values are means ± SD or the least square means (95% CI). *The least square means (95% CIs) were derived from ANCOVA adjusted for the baseline FMD, age, sex, current smoking, diabetes duration and BMI.

**Table 3:** **Changes in secondary efficacy measures in the sitagliptin and voglibose groups**

	**Sitagliptin**		**Voglibose**		**Between-group difference**
**Variable**	**mean ± SD**	**P value**	**mean ± SD**	**P value**	**P value**
HbA1C					
baseline at 0 W	7.04 ± 0.56		6.94 ± 0.45		
at 12 W	6.65 ± 0.57		6.59 ± 0.45		
12 W-0 W	−0.39 ± 0.60	0.0051	−0.35 ± 0.39	<0.0001	0.8021
GIP					
baseline at 0 W	289.92 ± 326.04		214.21 ± 220.15		
at 12 W	246.50 ± 284.25		258.41 ± 226.88		
12 W-0 W	−43.42 ± 190.10	0.3077	44.20 ± 198.54	0.2671	0.1323
GLP-1					
baseline at 0 W	5.71 ± 5.92		6.65 ± 10.18		
at 12 W	8.25 ± 6.37		6.66 ± 6.15		
12 W-0 W	2.54 ± 5.71	0.0611	0.00 ± 5.70	0.9973	0.1418
C-peptide					
baseline at 0 W	3.913 ± 2.567		3.037 ± 1.629		
at 12 W	3.909 ± 2.410		3.571 ± 2.710		
12 W-0 W	−0.003 ± 1.366	0.9906	0.534 ± 2.712	0.2977	0.3555
CD34					
baseline at 0 W	0.956 ± 0.563		0.896 ± 0.622		
at 12 W	1.134 ± 0.615		0.847 ± 0.470		
12 W-0 W	0.178 ± 0.379	0.0311	−0.050 ± 0.368	0.4656	0.0304
Total cholesterol					
baseline at 0 W	181.6 ± 28.8		187.8 ± 38.9		
at 12 W	172.5 ± 28.2		183.9 ± 46.6		
12 W-0 W	−9.0 ± 21.5	0.0509	−3.9 ± 30.0	0.4865	0.4807
Triglyceride					
baseline at 0 W	158.9 ± 131.1		141.2 ± 68.4		
at 12 W	122.4 ± 60.9		119.2 ± 51.9		
12 W-0 W	−36.5 ± 98.8	0.0837	−22.1 ± 52.0	0.0249	0.5217
HDL-C					
baseline at 0 W	56.4 ± 16.8		53.8 ± 13.0		
at 12 W	57.5 ± 24.4		50.3 ± 11.4		
12 W-0 W	1.0 ± 20.9	0.8096	−3.5 ± 8.3	0.0246	0.3223
adiponectin					
baseline at 0 W	13.80 ± 12.88		15.15 ± 13.81		
at 12 W	13.11 ± 11.10		14.09 ± 8.76		
12 W-0 W	−0.69 ± 2.71	0.2260	−1.06 ± 7.41	0.4413	0.8020
MDA-LDL					
baseline at 0 W	98.7 ± 32.0		112.4 ± 44.3		
at 12 W	99.7 ± 44.5		116.6 ± 49.7		
12 W-0 W	1.0 ± 33.8	0.8812	4.2 ± 45.0	0.6156	0.7787
8-OHdG					
baseline at 0 W	12.20 ± 6.55		12.31 ± 8.88		
at 12 W	13.10 ± 7.39		12.43 ± 15.46		
12 W-0 W	0.90 ± 7.30	0.5533	0.12 ± 17.29	0.9699	0.8252
hs-CRP					
baseline at 0 W	2194.4 ± 4079.1		2052.2 ± 3816.2		
at 12 W	1202.0 ± 1424.2		3322.7 ± 7813.6		
12 W-0 W	−992.4 ± 3609.6	0.1911	1270.5 ± 5592.1	0.2233	0.0783
PTX-3					
baseline at 0 W	1.606 ± 0.733		2.461 ± 2.989		
at 12 W	1.491 ± 0.585		1.848 ± 0.790		
12 W-0 W	−0.115 ± 0.462	0.2334	−0.613 ± 2.542	0.1967	0.3012
e-GFR					
baseline at 0 W	66.8 ± 20.8		63.6 ± 20.8		
at 12 W	62.2 ± 17.8		61.4 ± 19.9		
12 W-0 W	−4.6 ± 9.7	0.0301	−2.1 ± 7.7	0.1306	0.3007

GIP: gastric inhibitory peptide, GLP-1: glucagon-like peptide-1, HDL-C: high density lipoprotein cholesterol, MDA-LDL: malondialdehyde-modified low density lipoprotein, 8-OHdG: 8-hydroxy-2’-deoxyguanosine, hs-CRP: high-sensitivity C-reactive protein, PTX-3: pentraxin-3, e-GFR: estimated glomerular filtration rate. Data are expressed as mean ± SD.

### The primary endpoint

**Figure 2:**
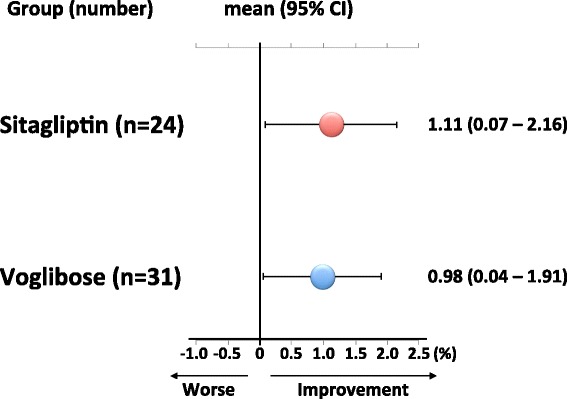
**Adjusted changes in FMD (ΔFMD) in the sitagliptin and voglibose groups.** Values are the least square means (95% CI). The least square means (95% CIs) were derived from ANCOVA adjusted for the baseline FMD, age, sex, current smoking, diabetes duration and BMI. ΔFMD was significantly improved after 12 weeks of treatment in both groups, and there was no significant difference in ΔFMD between the two groups.

Change in FMD (ΔFMD) was significantly improved after 12 weeks of treatment in both groups (sitagliptin: +1.11, 95% CI: 0.07-2.16 versus voglibose: +0.98, 95% CI: 0.04-1.91), and there was no significant difference in ΔFMD between the two groups (Table 2 and Figure 2).

### Secondary efficacy measures

**Figure 3:**
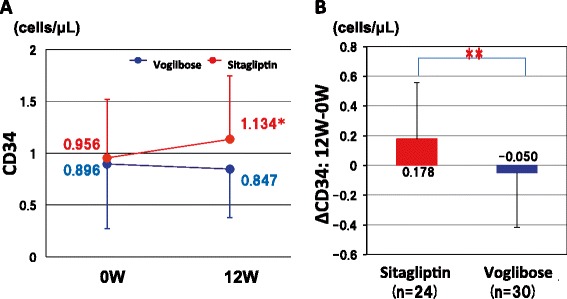
**Changes in CD34 in the sitagliptin and voglibose groups. A**. Circulating CD34 levels at 0 week and 12 weeks in the sitagliptin and voglibose groups. *P < 0.05 vs. 0 W, paired t-test. **B**. Change in CD34 from the baseline to the end of follow-up (ΔCD34). **P < 0.05, Student’s t-test.

There were no significant differences in changes in HbA1c, GIP, GLP-1, C-peptide, lipid profile, oxidative stress marker, inflammatory marker and eGFR between the two groups (Table 3). Sitagliptin tended to increase GLP-1 levels after 12 weeks of treatment (0 week: 5.71 ± 5.92 versus 12 weeks: 8.25 ± 6.37 pmol/L, P = 0.0611), but voglibose did not change the levels (0 week: 6.65 ± 10.18 versus 12 weeks: 6.66 ± 6.15 pmol/L, P = 0.9973). Compared with voglibose, sitagliptin significantly increased circulating CD34, a marker of positive endothelial progenitor cells (P < 0.05) (Table 3 and Figure 3).

### Adverse events

Adverse events were observed in 5 patients in the voglibose group (Table 4). There were no serious side effects in either group.

**Table 4:** **Adverse events**

**Events**	**Sitagliptin**	**Voglibose**
	**n = 29**	**n = 34**
Diarrhea	0 (0%)	1 (2.9%)
Nausea	0 (0%)	1 (2.9%)
Edema	0 (0%)	2 (5.9%)
Abdominal fullness	0 (0%)	1 (2.9%)

Number of cases (%) is shown.

## Discussion

In summary, ΔFMD was significantly improved after 12 weeks of treatment in both the sitagliptin and voglibose groups, and there was no significant difference in ΔFMD between the two groups.

Alpha GI improves postprandial endothelial dysfunction in patients with type 2 diabetes [9,10]. We showed that not only voglibose, an alpha GI, but also sitagliptin, a DPP-4 inhibitor, improves endothelial function as assessed by FMD in patients with type 2 diabetes. The impact of a DPP-4 inhibitor on endothelial dysfunction in patients with type 2 diabetes is controversial. A recent study showed that sitagliptin improved endothelial dysfunction assessed by the reactive hyperemia arterial tonometry index (RHI) after 6 months of treatment in patients with diabetes and coronary artery disease [24]. We also showed that sitagliptin improved endothelial dysfunction assessed by another method, FMD. A prospective observational single arm trial also showed that the treatment with sitagliptin for 12 weeks increased FMD in patients with type 2 diabetes [36]. Here, we conducted a randomized prospective multicenter study in patients with type 2 diabetes and revealed improvement of FMD after the 12-week treatment with sitagliptin. In contrast, another short-term study showed that DPP-4 inhibitors, including sitagliptin or alogliptin, attenuated endothelial function as evaluated by FMD after 6 weeks of treatment in patients with diabetes [19]. Furthermore, voglibose, an alpha GI, did not affect FMD in the study. Therefore, short-term treatment with either DPP-4 inhibitors or alpha GI does not ameliorate endothelial dysfunction in patients with diabetes. Our study showed that 12-week treatment with either sitagliptin or voglibose ameliorated endothelial function. Thus, at least 12 weeks of treatment is needed to improve endothelial function.

The STOP-NIDDM trial showed that an alpha GI reduces the risk of cardiovascular events in patients with IGT [12]. DPP-4 inhibitors are also expected to have beneficial cardiovascular effects [37,38]. However, two recent trials with DPP-4 inhibitors, SAVOR-TIMI 53 and EXAMINE trials, showed that saxagliptin and alogliptin did not increase or decrease ischemic events in patients with type 2 diabetes [39,40]. These trials demonstrated the safety of DPP-4 inhibitors but did not demonstrate cardiovascular benefits. Patients at high risk patients for cardiovascular disease were enrolled in those studies. In the SAVOR-TIMI 53 trial, about 78% of the patients had established cardiovascular disease, and the EXAMINE trial consisted of patients with acute coronary syndrome. Mean HbA1c levels were about 8.0 in patients of those studies. Our study included relatively low-risk patients. Only about 25% of the patients in our study had established cardiovascular diseases. Mean HbA1c levels were about 7.0. ΔFMD was significantly improved after 12 weeks of treatment in both the sitagliptin and voglibose groups in our study. The clinical implication of this study is the demonstration of beneficial effects of both sitagliptin and voglibose on endothelial dysfunction in low-risk and mild-risk patients with type 2 diabetes. Therefore, sitagliptin and voglibose might have cardiovascular benefits in low-risk and mild-risk patients with type 2 diabetes. Further studies are needed to clarify the long-term benefits for cardiovascular disease [37].

Iwamoto et al. reported that once-daily sitagliptin monotherapy (50 mg/day) showed greater efficacy and better tolerability than did thrice-daily voglibose (0.6 mg/day) over 12 weeks in Japanese patients with type 2 diabetes [41], but there was no significant difference in changes in HbA1c between the sitagliptin (50 mg/day) and voglibose (0.6 mg/day) groups in our study. Mean HbA1c levels at baseline in the former study and our study were about 7.8 and 7.0, respectively. Lower HbA1c at baseline in our study might have had an influence on changes in HbA1c levels. Sitagliptin tended to increase GLP-1 levels after 12 weeks of treatment, but there was no significant difference in changes in GIP and GLP-1 between the two groups in this study. These results might be because of the small sample size.

Matsubara et al. reported that treatment with sitagliptin for 6 months improved endothelial dysfunction along with a decrease in hs-CRP in patients with coronary artery disease and uncontrolled diabetes [24]. Lipid profiles and eGFR did not change after treatment with sitagliptin in that study as well as in our study. Our study showed no significant difference in changes in hs-CRP after treatment with sitagliptin. Furthermore, Sakamoto et al. reported that sitagliptin improved blood pressure and lipid profiles in patients with type 2 diabetes [42]. These different results might be due to the small sample size, short-term treatment or study in low risk patients with type 2 diabetes.

Fadini et al. reported that sitagliptin increased circulating EPCs in type 2 diabetic patients with concomitant upregulation of stromal-derived factor-1α (SDF-1α) [21]. SDF-1α is a chemokine that stimulates bone marrow mobilization of EPCs and is one of the substrates of DPP-4. We also showed that sitagliptin significantly increased circulating CD34, but voglibose did not increase CD34. Therefore, this is an ancillary effect of sitagliptin and it is independent from blood glucose levels. Liao et al. reported that the number of EPCs was an independent risk factor for FMD [20]. Thus, this effect might be favorable for the preservation of endothelial function in the future. Further long-term studies are needed to clarify this point.

A recent study revealed that alogliptin, a DPP-4 inhibitor, did not increase the frequency of serious adverse events, including hypoglycemia, cancer, pancreatitis, and initiation of dialysis in patients with type 2 diabetes who had a recent acute coronary syndrome [40]. A pooled analysis of 25 randomized clinical trials did not indicate that treatment with sitagliptin increases cardiovascular risk in patients with type 2 diabetes mellitus [43]. Our study also showed that sitagliptin did not cause serious adverse events. Thus, DPP-4 inhibitors may be safe drugs for patients with type 2 diabetes.

### Study limitations

The first limitation of this study was the small sizes of the groups. The second was heterogeneity of the population in this study. The age range was quite wide in our study. The third limitation was that our study was a short-term study. Our findings need to be confirmed in a large cohort of patients with type 2 diabetes and with a long period.

In conclusion, sitagliptin improves endothelial dysfunction just as well as voglibose in patients with type 2 diabetes. Furthermore, sitagliptin increases the level of circulating CD34. Sitagliptin has protective effects on endothelial function without adverse events. These effects have potential favorable cardiovascular implications for patients with type 2 diabetes.

## Abbreviations

BMI: Body mass index
DPP-4: Dipeptidyl peptidase IV
eGFR: Estimated glomerular filtration rate
FMD: Flow-mediated dilatation
GIP: Gastric inhibitory peptide
GLP-1: Glucagon-like peptide-1
GI: Glucosidase inhibitor
hs-CRP: High-sensitivity C-reactive protein
8-OHdG: 8-hydroxy-2’-deoxyguanosine
IGT: Impaired glucose tolerance
MDA-LDL: Malondialdehyde-modified low density lipoprotein
PTX-3: Pentraxin-3
PPH: Postprandial hyperglycemia
